# Sequence to structure insights into Lassa virus population-level biophysical properties and glycoprotein structure catalogue

**DOI:** 10.1038/s44298-026-00196-3

**Published:** 2026-05-14

**Authors:** Richard Olumide Daodu, Jakob R. Riccabona, Antonia Sophia Peter, Jens-Uwe Ulrich, Isabel von Creytz, Joseph B. Prescott, Knut Reinert, Clara T. Schoeder, Denise Kühnert

**Affiliations:** 1 Center for Artificial Intelligence in Public Health Research, Robert Koch Institute, Berlin, Germany; 2 Department of Mathematics and Computer Science, Freie Universität Berlin, Berlin, Germany; 3 Institute for Drug Discovery, Faculty of Medicine, Leipzig University, Leipzig, Germany; 4 Center for Scalable Data Analytics and Artificial Intelligence ScaDS.AI, Dresden/Leipzig, Leipzig, Germany; 5 Center for Biological Threats and Special Pathogens, Robert Koch Institute, Berlin, Germany; 6 Max-Planck-Institute for Molecular Genetics, Berlin, Germany

**Keywords:** Computational biology and bioinformatics, Microbiology

## Abstract

Lassa virus (LASV) remains a major public health threat in West Africa, with recurrent outbreaks, exported cases, and no licensed vaccine. LASV lineages are geographically separated and differ in immunogenicity and pathogenicity; however, the fundamental biophysical properties that may explain these differences remain poorly defined. Here, we analyse LASV protein properties at the population scale across lineages, focusing on the glycoprotein (GP), the principal target of humoral immunity. Across hundreds of curated sequences, protein length variation is driven primarily by short indels, with pronounced variation in the RNA polymerase and a recurrent one-amino-acid difference in GP. In parallel, population-scale analyses reveal subtle lineage- and protein-specific differences in amino-acid composition across the LASV proteins. Despite co-circulation in Nigeria, S-segment–encoded proteins from lineage III are consistently heavier than those from lineage II. An integrative framework combining random forest feature importance, Manhattan-distance profiling, Pearson correlation, and amino-acid composition analyses reveals that lineage III GPs are ~180 Da heavier on average, driven by shifts toward the use of heavier residues at specific sites. Population-scale computational structural modelling and flow-cytometric assays indicate that the N-terminal GP1 indel is structurally and functionally tolerated. Together, these findings define lineage-specific biophysical patterns in LASV and provide a catalogue of GP structures to inform vaccine and therapeutic design.

## Introduction

Lassa fever, an acute haemorrhagic disease caused by Lassa virus (LASV), remains a persistent public health challenge, with an estimated 100,000–300,000 infections and up to 5000 deaths annually in West Africa^1^. LASV is a single-stranded RNA virus from the *Arenavirus* family and is increasingly recognised as a global threat, as evidenced by multiple imported cases world wide^2^ and rare but documented secondary transmission events in Europe^3^. Highlighting the risk of further geographic spread, an imported case was recently reported in China^4^. Meanwhile, climate change has been reported to potentially expand the endemic range of LASV in the coming years^5^. Despite more than five decades of research, there is still no licensed vaccine and no specific antiviral approved for Lassa fever. Ribavirin remains the only widely used treatment, yet its clinical benefit and optimal application remain uncertain^6,7^. This combination of high endemic burden, exportation risk, and limited countermeasures makes it urgent to study LASV in ways that can further explain its biology and directly inform drug and vaccine development.

The LASV genome comprises two RNA segments, Small (S) and Large (L), encoding four essential proteins: the glycoprotein complex precursor (GPC), nucleoprotein (NP), matrix protein (Z), and RNA-dependent RNA polymerase (L)^6^. GPC is expressed as a single polyprotein that is cleaved into the stable signal peptide (SSP), the receptor-binding subunit GP1, and the fusion subunit GP2^8^. As the sole surface-exposed protein, GPC mediates host-cell entry and is the primary target for neutralizing antibodies, small-molecule entry inhibitors, and most vaccine candidates^6,9,10^.

To date, genomic analyses have defined seven major LASV lineages with largely distinct geographic distributions: lineages I–III in Nigeria^11^, lineage IV in Sierra Leone, Guinea, and Liberia^12^, lineage V in Mali and Côte d’Ivoire^13^, and lineage VII in Togo and Benin^14,15^; a rodent-associated strain from *Hylomyscus pamfi* in Nigeria has been designated lineage VI^16^. Beyond their geographic separation, these lineages also differ in clinical severity and immunological responses. In non-human primates, recent work suggests that lineage VII strains may cause more severe disease than lineage II^17^, and findings from monoclonal antibody therapy studies also suggest increased virulence of lineage VII^18^. Lineage I shows resistance to many known neutralizing antibodies^19^, and additionally, survivor plasma from patients appears to exhibit lineage-biased neutralization^20,21^. Together, these observations imply that lineage-specific molecular features, beyond nucleotide divergence, may contribute to differences in virulence and immune recognition. However, which physical or structural properties distinguish these lineages, and whether such properties are detectable at a population scale, remain poorly defined.

LASV shows substantial diversity both between and within lineages at the nucleotide and amino-acid levels (~25–30% nucleotide divergence and 10–15% amino-acid divergence)^12,22^, giving rise to defined sublineages^11^. Most studies of LASV diversity have focused on phylogenetic reconstruction and molecular epidemiology, elucidating lineage structure, geographic spread, evolutionary history, and host adaptation^11,12,22,23^. While these approaches have been essential for defining lineages and identifying signatures of selection, they rarely translate sequence variation into basic protein-level descriptors at a population scale. These descriptors include fundamental biophysical properties such as protein length, molecular mass, amino-acid composition, and charge, which define the physical state of viral proteins as they exist in nature. Establishing the natural distributions of these properties provides a baseline for interpreting historical variation and enables the detection of future deviations, including the emergence of novel variants or atypical protein features that may warrant functional or public-health attention. Protein function is mediated through biophysical properties that depend on fold, surface features, post-translational modifications, and dynamics. Even small mass differences (1–100 Da), arising from amino-acid substitutions or modifications such as deamidation or phosphorylation, can alter charge, interactions, activity, or localisation and are detectable by mass spectrometry^24,25^. For LASV, biophysical properties have typically been reported for single reference strains, vaccine constructs, or limited sequence panels^26,27^, providing only a narrow view of the diversity present in nature. Meanwhile, although codon usage bias has been characterised in LASV^12,27^, there remains a need to elucidate lineage-resolved amino-acid composition and protein-level properties.

Population-scale analyses of LASV proteins remain scarce and are largely confined to the glycoprotein (GPC), where recent studies have primarily focused on amino-acid substitutions, epitope variation, or evolutionary rates^23,28^. Other fundamental, lineage-resolved protein descriptors—such as length variation, mass distributions, or compositional shifts—have been largely overlooked. This gap is particularly evident for insertions and deletions (indels), which are a major cause of protein length variation and an important source of genomic and evolutionary diversity^29^. Although lineage-associated indels and alignment gaps in GPC have been noted near GP1 epitopes–around amino acid position 60^19,22,23,30,31^, their prevalence, population-level patterns, and functional consequences have not been systematically examined. Notably, even recent deep mutational scanning studies have focused almost exclusively on substitutions and have not experimentally addressed indels^32^. Despite the rapid expansion of available LASV genomic data in recent years^11,12,33,34^, lineage-resolved analyses of protein length variation and associated biophysical properties remain largely unexplored.

In this study, to provide a clearer understanding of LASV diversity, we perform an analysis of the theoretical biophysical properties of all four LASV proteins, with particular emphasis on GPC. We quantify sequence length and theoretical molecular mass across all curated LASV protein sequences in GenBank, exploring whether lineages exhibit consistent shifts in these features. We apply machine-learning and mathematical approaches to identify amino-acid composition patterns that distinguish lineages. As part of our analysis of the lineage-specific indel at GPC amino acid position 60/61, which we find to occur on a population level, we predict structures for all available GPC sequences of appropriate quality using state-of-the-art structure-prediction pipelines, thereby generating, to our knowledge, the first structural catalogue of LASV glycoprotein models (10.5281/zenodo.17642142). Further, we provide evidence for the structural tolerance of the GP1 N-terminal indel based on expression and antibody binding experiments. Together, these analyses provide a molecular and structural framework for understanding lineage-specific LASV diversity and may guide rational vaccine and therapeutic design.

## Results

### Lassa virus proteins differ in protein molecular mass and length across lineages

**Fig. 1:**
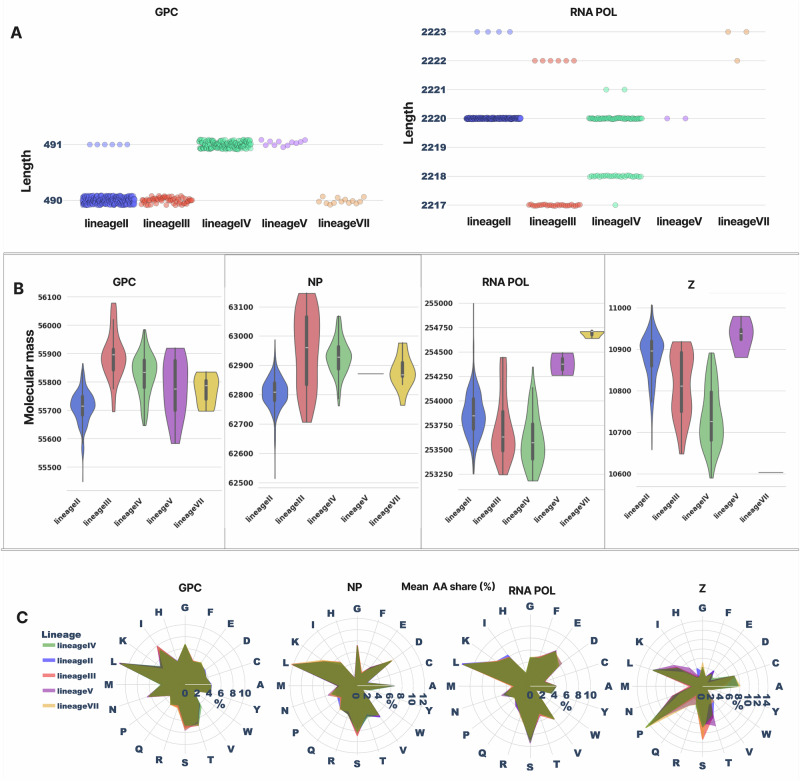
Lassa Virus proteins differ in length, molecular mass, and amino acid composition across lineages. **A** Protein length for each LASV protein (x-axis), coloured by lineage. For GPC, two major length classes are evident: 490 aa (lineages II, III, and VII) and 491 aa (lineages IV and V), with a small subset of 491-aa sequences within lineage II. For NP, no difference in protein length can be found. The RNAPOL can be seen to be very variable in length across lineages. **B** Molecular Mass differences for each LASV protein are represented as violin plots with mean and error bars represented as boxes. **C** Radar plots of mean amino acid composition for each lineage, suggesting subtle differences in amino acid usage for all proteins except for the Z protein. Overall, the colours blue, red, green, purple, and yellow represent lineages II, III, IV, V, and VII, respectively, and this colour scheme was maintained consistently across most figures, with exceptions (Fig. 4A, B) where alternative colouring was used for clarity.

To gain a comprehensive overview of protein-level differences across LASV lineages, we quantified fundamental properties, such as protein length, molecular mass, and amino acid composition for all LASV proteins (Fig. 1). Our analysis revealed several lineage-specific patterns, with the GPC and the Z protein showing the most striking differences. Due to limited sequence availability, lineages I and VI were excluded from this analysis (see Methods).

**Fig. 2:**
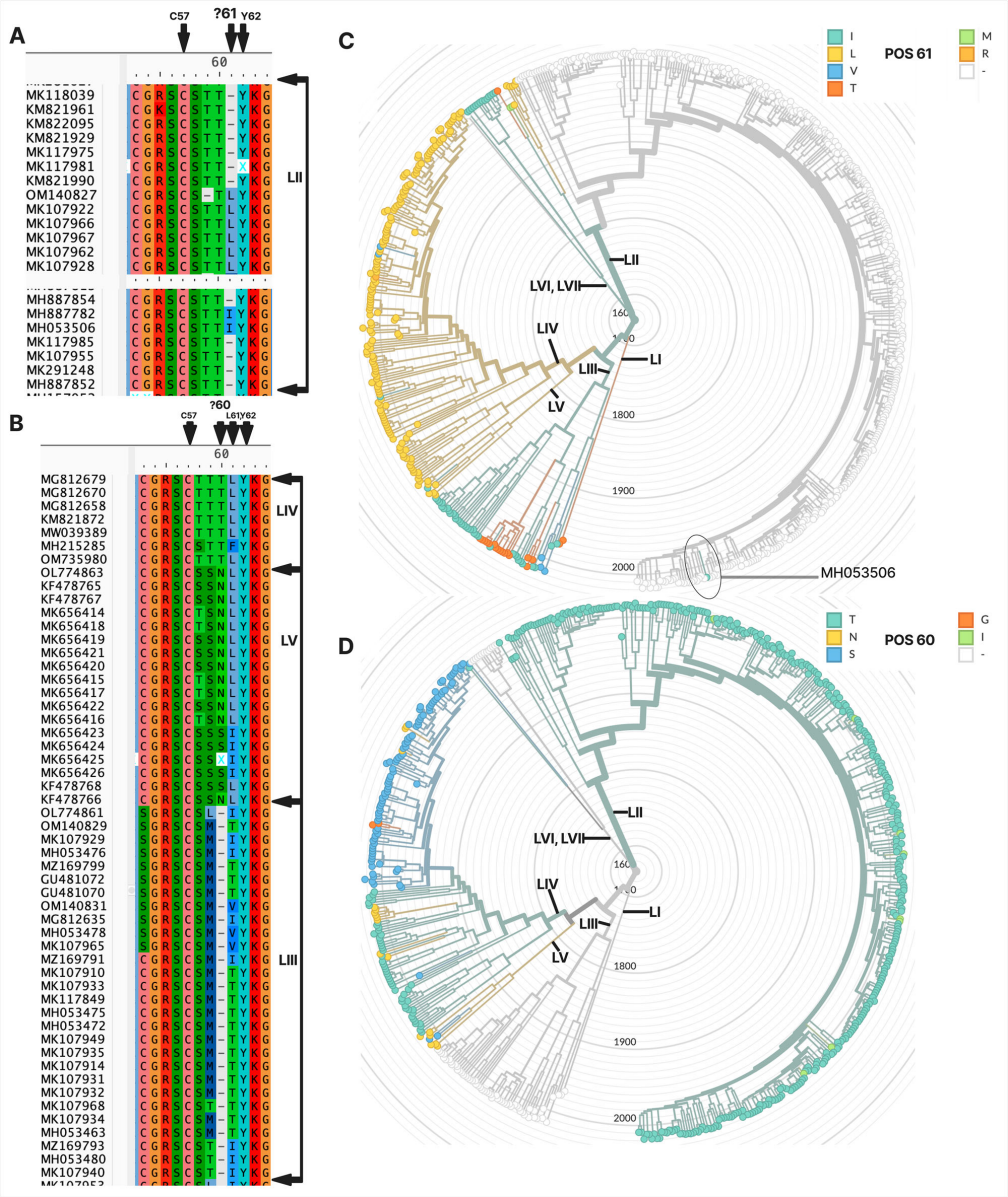
Indel around GPC residue 60/61 underlies lineage-specific length polymorphism. **A** Local amino-acid alignment of LASV GPC sequences from lineage II (LII) spanning residues C57–Y62. Most LII sequences lack a residue at position 61 (shown as a gap), producing a 490-aa GPC. A minority of LII sequences (e.g., MK107922, MK107966, MK107967, MK107962, MK107928) encode leucine at this position (L61), corresponding to a 491-aa GPC and supporting the existence of both length variants within the lineage. OM140827 shows a gap at position 59; however, this gap can be reasonably placed at neighbouring positions without substantially altering local alignment quality, illustrating the residual ambiguity in this region. **B** Equivalent alignment window for lineages IV (LIV) and III (LIII). In LIV and LV, L61 is almost universally present, whereas LIII and other lineages show alternative residues and occasional gaps in this region. Cys57 and Tyr62 are highly conserved in all lineages and serve as anchors for positional homology, while residues at positions 60–61 show lineage-specific variation consistent with an amino-acid indel. **C** Maximum-likelihood phylogeny of LASV GPC sequences displayed in radial format, with tips coloured by the amino acid present at position 61 (legend, top right). Major lineages are indicated on the tree (LI, LII, LIII, LIV, LV, LVI/LVII). There is no residue at position 61 in most LII sequences (gap), whereas other lineages predominantly encode isoleucine, leucine, valine, or threonine at this position. The outlying sequence MH053506 (circled) carries a residue at this site despite being nested within gap-containing LII sequences, consistent with a rare, isolate-specific indel event. **D** Radial phylogeny as in (**C**) but with tips coloured by the amino acid at position 60 (legend, bottom right). Variation at this site is dominated by threonine, asparagine, and serine, with occasional glycine or isoleucine, again showing lineage-structured patterns. Together, **C** and **D** indicate that a short indel centred on residues 60–61—rather than widespread alignment or sequencing errors—most likely explains the observed 490/491-aa GPC length polymorphism across LASV lineages.

For the GPC, two predominant lengths were observed (Fig. 1A): 491 amino acids for lineages IV and V, and 490 amino acids for lineages II, III, and VII. This pattern suggests that the previously reported alignment gaps in GPC^19,22,23,30,31^ reflect length differences at a population scale. Notably, 5 out of 396 sequences from lineage II had a length of 491 residues (subsequently investigated in Fig. 2). We next examined whether these length differences were associated with changes in molecular mass by calculating the theoretical mass of each GPC sequence and then estimating the mean mass per lineage. Intriguingly, despite being shorter by one amino acid than lineages IV and V, lineage III GPC was the heaviest (Fig. 1B). Ranked by mean molecular mass, lineage III had the highest mean of 55,891.90 Da (*n* = 56; SD = 83.78), followed by lineage IV at 55,825.04 Da (*n* = 165; SD = 72.86), lineage V at 55,778.45 Da (*n* = 11; SD = 105.11), and lineage VII at 55,771.76 Da (*n* = 13; SD = 51.57). Lineage II had the lowest mean mass at 55,711.53 Da (*n* = 396; SD = 57.84). These values correspond to the monomeric GPC; for the biological trimeric state of LASV GPC, the masses would simply be three times higher. To explore the basis for this mass discrepancy, we analyzed the amino acid composition (AAC) of each sequence and then averaged AAC per lineage (Fig. 1C). All lineages (GPC) showed leucine as the most abundant amino acid, consistent with its general prevalence in proteins^34^. To determine whether subtle AAC differences were informative, we trained a simple random forest classifier using per-sequence AAC to predict lineage affiliation. The model performed well (see associated GitHub workflow), with an accuracy of 0.951, demonstrating that although amino-acid usage appears visually similar (Fig. 1C), compositional profiles are sufficiently distinct to allow highly accurate classification.

We next focused on the NP, which was found to have a length of 569 amino acids across all lineages and all samples. Despite this fully conserved length, lineage III again had the highest mean molecular mass, at 62,952.35 Da (*n* = 55, SD = 130.21), followed closely by lineage IV at 62,928.48 Da (*n* = 155, SD = 59.51) (Fig. 1B). Lineage VII had a mean mass of 62,879.86 Da (*n* = 13, SD = 57.62), and lineage V 62,871.45 Da based on a single sequence (*n* = 1; SD is not reported because the sample size was too low, but we retained this value for completeness). Lineage II was the lightest, with a mean of 62,806.78 Da (*n* = 410, SD = 53.49). Thus, even with identical protein length, lineage III NP is ~140 Da heavier than lineage II NP, reinforcing the pattern already observed for GPC. Together, these findings indicate that lineage III encodes consistently heavier proteins than lineage II on the S segment of the genome, with average mass increases of ~180 Da in GPC and ~140 Da in NP relative to lineage II (Fig. 1B). The specific positions and underlying amino acids responsible for these patterns are examined in subsequent analyses. The consistent NP length of 569 amino acids contrasts with the 570-aa NP reported for the historical strain GA391 (lineage III)^22,35^. The GA391 sequence did not pass our curation filters and was therefore excluded from the final dataset; all remaining lineage III NP sequences in GenBank that met our quality criteria are 569 aa in length. This discrepancy may reflect a sequencing or annotation artefact in the original report, or a rare length variant that is not represented among currently circulating viruses. In either case, the difference between a single historical reference and hundreds of contemporary sequences underscores the value of population-scale analyses for accurately defining LASV protein properties across lineages. In summary, our data indicate that LASV NP is 569 amino acids long across all lineages, and not only in the Josiah reference strain (NP_694869.1).

We next examined the L-segment proteins, the RNA-dependent RNA polymerase (RNAPOL) and the Z matrix protein. RNAPOL lengths were tightly clustered, varying by up to six amino acids overall (2,217–2,223 aa), but displayed clear lineage-associated patterns (Fig. 1A; Table 1). The 2,220-aa isoform predominated overall and was the dominant form in lineages II, IV, and V (e.g., 287/291 [98.6%] in lineage II). In contrast, lineage III lacked 2,220-aa sequences and was enriched for the shorter 2,217-aa form (26/32 [81.3%]), whereas lineage VII contained only longer RNAPOL variants (2,222–2,223 aa), making it the lineage with the longest polymerase on average and lineage III the shortest. Lineage IV was comparatively heterogeneous, with a major 2,220-aa class alongside a substantial 2,218-aa class (Table 1). The functional consequences of the underlying indels remain unclear; however, the LASV RNAPOL has been implicated as a determinant of pathogenic variability between clinical isolates in guinea pig models^36^.

**Table 1:** LASV protein length and theoretical molecular mass by lineage (with Δ from lineage IV)

Protein	Lineage	n	Length range (aa)	Length distribution (aa: count (%))	Mean mass (Da)	SD (Da)	Mean mass Δ from IV (Da)
GPC	II	396	490–491	490 aa: 391 (98.74%) 491 aa: 5 (1.26%)	55,711.53	57.84	113.51
GPC	III	56	490	490 aa: 56 (100.00%)	55,891.90	83.78	-66.86
GPC	IV	165	491	491 aa: 165 (100.00%)	55,825.04	72.86	0.00
GPC	V	11	491	491 aa: 11 (100.00%)	55,778.45	105.11	46.59
GPC	VII	13	490	490 aa: 13 (100.00%)	55,771.76	51.57	53.28
NP	II	410	569	569 aa: 410 (100.00%)	62,806.78	53.49	121.70
NP	III	55	569	569 aa: 55 (100.00%)	62,952.35	130.21	-23.87
NP	IV	155	569	569 aa: 155 (100.00%)	62,928.48	59.51	0.00
NP	V	1	569	569 aa: 1 (100.00%)	62,871.45	NaN	57.03
NP	VII	13	569	569 aa: 13 (100.00%)	62,879.86	57.62	48.62
RNAPOL	II	291	2,220–2,223	2,220 aa: 287 (98.63%) 2,223 aa: 4 (1.37%)	253,857.50	226.70	-264.72
RNAPOL	III	32	2,217–2,222	2,217 aa: 26 (81.25%) 2,222 aa: 6 (18.75%)	253,730.65	337.42	-137.87
RNAPOL	IV	84	2,217–2,221	2,220 aa: 54 (64.29%) 2,218 aa: 27 (32.14%) 2,221 aa: 2 (2.38%) 2,217 aa: 1 (1.19%)	253,592.78	264.13	0.00
RNAPOL	V	2	2,220	2,220 aa: 2 (100.00%)	254,374.63	162.11	-781.85
RNAPOL	VII	3	2,222–2,223	2,223 aa: 2 (66.67%) 2,222 aa: 1 (33.33%)	254,685.44	41.04	-1,092.66
Z	II	335	98–99	99 aa: 334 (99.70%) 98 aa: 1 (0.30%)	10,883.30	55.56	-146.91
Z	III	33	99	99 aa: 33 (100.00%)	10,815.45	77.60	-79.06
Z	IV	116	98–100	99 aa: 114 (98.28%) 98 aa: 1 (0.86%) 100 aa: 1 (0.86%)	10,736.39	72.70	0.00
Z	V	4	99	99 aa: 4 (100.00%)	10,933.93	40.70	-197.54
Z	VII	4	99	99 aa: 4 (100.00%)	10,603.23	0.00	133.16

In contrast, Z protein length was highly conserved across lineages: more than 99% of sequences encoded a 99-aa protein, with only three exceptions. Two sequences, OM140826 (lineage II) and KM821825 (lineage IV), encoded 98-aa variants, while one sequence, MH215282 (lineage IV), encoded a 100-aa variant (Table 1). It is unclear whether these rare length differences represent true biological variation or sequencing or annotation artefacts. Overall, whereas lineage III consistently exhibited greater molecular mass than lineage II for S-segment proteins (GPC and NP), L-segment patterns were more variable, with lineage V encoding the heaviest Z protein and lineage VII encoding the heaviest RNAPOL.

Although there are visually apparent but generally subtle differences in amino-acid composition between lineages (Fig. 1C), the lineage profiles for each protein largely overlap, especially for GPC, NP, and RNAPOL. Instead, the dominant signal is protein- and segment-specific composition. Among S-segment proteins, based on the top five ranked amino acids, GPC is dominated by L, I, S, T, and G, whereas NP is enriched for L, S, D, K, and G, with D ranking among the top residues in NP but not in GPC. For the L-segment, RNAPOL shows a similar hydrophobic/charged profile across lineages, with L, E, K, S, and V among the most abundant residues. In contrast, the Z matrix protein is compositionally distinct, with a strong enrichment for proline and more pronounced lineage-to-lineage variation in its radar profile, particularly for lineages V and III. Overall, lineage VII uses the most proline (15.15% of residues), followed by lineages IV (15.03%), V (14.14%), III (13.69%), and II (11.71%). Proline-rich late-domain motifs in LASV Z (PTAP and PPPY, a PPxY-type motif) are known to be critical for virus-like particle (VLP) budding, and mutations or deletions that disrupt these motifs substantially reduce VLP release in cell-based assays^37–40^. Whether the lineage-specific differences in overall proline usage that we observe translate into measurable differences in Z-mediated budding or egress remains an open question. Consistent with global proteome trends^41^, tryptophan (W) is the least used amino acid across all LASV proteins. These segment- and protein-specific differences in amino-acid usage, superimposed on relatively minor lineage effects, likely contribute to the variation in molecular mass that we observe across proteins and lineages.

### N-terminal indel near GPC 60/61 defines lineage-specific length variation

Seeing the widespread variation in the physical properties of LASV proteins, we next focused on identifying the cause of length variation in GPC. Accurate inference of lineage-specific mutations depends critically on the quality of the underlying multiple sequence alignment. Multiple sequence alignment is particularly challenging for LASV, as demonstrated by the range of different placements of the GPC indel reported in previous work^19,22,23,30,31^. Mutation-level and population-scale analyses impose strict requirements: each alignment column must represent truly homologous positions across all sequences. If residues are misaligned, apparent “diversity” at a given site may be spurious, and downstream applications—such as selecting positions for functional assays or designing mutagenesis experiments—may be misdirected or may even unintentionally generate viruses that do not exist in nature.

**Fig. 3:**
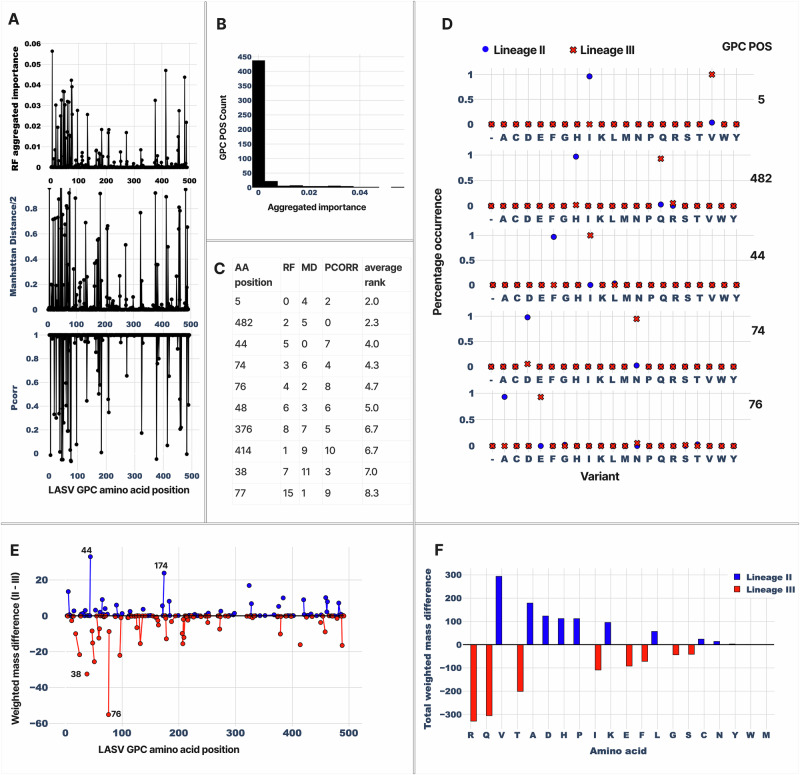
Lineage III GPC is heavier than lineage II because of a shift in amino-acid usage. **A** Population-scale comparison of lineage II and lineage III GPC sequences using three complementary metrics along the alignment: random-forest (RF) aggregated importance (top), Manhattan distance/2 (middle), and Pearson correlation (bottom). Peaks indicate positions where amino-acid usage differs systematically between the two lineages. **B** Distribution of RF aggregated importance values across all GPC positions. Only 147 of 491 sites have non-zero importance, indicating that more than half of GPC is effectively indistinguishable between the two lineages. **C** Top-ranked positions based on the average rank of the three metrics (PCORR, RF, MD), with amino-acid position and individual ranks shown in the table. **D** Amino-acid distributions at selected high-ranking positions (e.g., 5, 482, 44, 74, 76). For each position, the percentage occurrence of each residue is shown for lineage II (blue) and lineage III (red), highlighting strongly lineage-biased variants, including the immunologically relevant site 76. **E** Left: per-position weighted molecular mass difference (lineage II − lineage III) along GPC. Positive values (blue) indicate positions where lineage II is heavier; negative values (red) indicate positions where lineage III is heavier (I77 positions are non-zero in total). Positions 44 and 76 are among the largest contributors in favour of lineages II and III, respectively. Slanted lines indicate consecutive positions. **F** Right: total weighted mass contribution of each amino acid across all positions, showing that increased usage of relatively heavier residues (e.g., R, Q, T, I, E, F) in lineage III and of lighter aliphatic residues (e.g., V, A, L, P) in lineage II underlies the global mass shift between the two lineages. Slanty lines indicate consecutive positions.

Because GPC length segregates almost entirely into 490- and 491-amino-acid forms, we sought to pinpoint the residues responsible for this difference and to determine whether it reflects a genuine biological indel or an artefact of alignment or sequencing. We realigned all GPC sequences at the amino-acid level and focused on the N-terminal region where the indel appeared to reside. Using the conserved cysteine 57 (C57) and tyrosine 62 (Y62) as anchors, we localized the length polymorphism to the short interval spanning positions 57–62, which lies around the cleavage site between the stable signal peptide (SSP) and GP1 (Fig. 3A, B). C57 and Y62 are fully conserved across all lineages, strongly supporting the homology of this window and arguing against gross misalignment.

Although this region has rarely been examined at the population scale, previous work has noted a gap near position 60 in selected lineage II, III, and VII sequences^19,22,23,30,31^. Here, we characterize this polymorphism comprehensively. Within the 57–62 interval, most lineage II sequences are characterized by a gap at position 61, whereas lineage IV and most other lineages encode a hydrophobic residue at this site—most frequently leucine, but also isoleucine, valine, or threonine (Fig. 2A, B). Thus, the canonical 490-aa lineage II GPC can be interpreted as carrying an in-frame indel at position 61 relative to the 491-aa form. This interpretation is supported by the fact that a minority of lineage II sequences (e.g., MK107922, MK107966, MK107967, MK107962, MK107928) encode leucine at this position (L61), matching the prevalent residue at this site in other lineages, and have a total length of 491 amino acids. These “long” lineage II sequences cluster within a small subclade (sublineage 2A or 2B as defined by Ehichioya et al.^11^) of the broader lineage II phylogeny (Fig. 2C), suggesting that they represent genuine biological variants on a typical lineage II background rather than systematic sequencing or annotation errors. In this sense, our population-scale analysis refines earlier descriptions that focused on a few representative sequences with a gap at position 60 in lineage II^19,30,31^, suggesting that the indel may be in fact in position 61.

Phylogeny-aware mapping of the amino acid present at position 61 reveals that hydrophobic residues (I, L, V, T) at this site are common in non–lineage II clades, whereas the gap state is concentrated in most lineage II sequences (Fig. 2C). One lineage II sequence, MH053506, appears as an outlier on the tree: it carries a residue at position 61 despite being nested within gap-containing lineage II sequences. This pattern is consistent with a rare, isolate-specific indel event that reintroduces an amino acid at this position - likely an insertion event. However, because this observation is based on a single sequence from one laboratory, we cannot exclude the possibility of a technical or annotation error.

Lineages III and VII illustrate the residual uncertainty that remains in this highly variable region. For these lineages, the aligner placed the indel at position 60 rather than 61. Visually, some residues currently aligned at position 61 could reasonably be shifted to position 60 without substantially degrading alignment quality, and alternative gapping schemes would slightly shift the inferred position of the indel. We therefore regard the exact register of residues 60 and 61 in these lineages as somewhat ambiguous. Nevertheless, all reasonable alignments support the same qualitative conclusion: the GPC 490/491-aa polymorphism is driven by a short N-terminal indel in the 60–61 region. This local ambiguity also frustrates definitive reconstruction of the underlying evolutionary event—whether the gap in lineage II arose from a deletion, whether other lineages gained an insertion, or whether both processes occurred at different times.

### Lineage III GPC is heavier than lineage II because of a shift in amino-acid usage

Lineages II and III both circulate in Nigeria, albeit in largely non-overlapping regions^11^ and their GPCs are almost always of the same length (490 amino acids), with only a small subset of 491-aa sequences in lineage II (Fig. 1). Yet lineage III GPC is consistently heavier than lineage II, and indeed is the heaviest GPC across all lineages, even though it is one amino acid shorter than lineages IV and V due to the N-terminal indel at position 60/61. We therefore investigated which specific residues and positions drive the molecular mass difference between lineages II and III.

To identify where the two lineages differ on a population scale, we applied three complementary metrics to the GPC alignment: random forest (RF)–derived aggregated feature importance, Manhattan distance, and Pearson correlation (Fig. 3A). All three profiles highlight a similar set of discrete regions along GPC, indicating that only a subset of positions shows systematic lineage-specific differences (Fig. 3A). RF aggregated importance further revealed that only 147 of 491 alignment positions have any non-zero contribution at all (Fig. 3B), implying that more than half of GPC is essentially conserved, or at least identically distributed, between the two lineages. We then combined the three metrics into an average rank to prioritise positions for follow-up (Fig. 3C). The top-ranked sites include positions 5, 38, 44, 74, 76, 77, 376, and 414. Inspection of the amino-acid distributions at these positions confirms pronounced lineage specificity (Fig. 3D), with different residues dominating in lineages II and III. Particularly noteworthy is position 76, which shows almost completely distinct variants between the two lineages and has previously been implicated as an immunologically important site in LASV GPC^32^.

To quantify how much each position contributes to the overall mass difference, we computed the per-position weighted mass difference (lineage II − lineage III) by multiplying the difference in amino-acid frequencies at each site by the residue masses and summing over all amino acids. This analysis shows that most positions make only small contributions, but a few sites stand out as major drivers (Fig. 3E). Position 76 is the single largest contributor in favour of lineage III (large negative peak), whereas position 44 and a small number of other sites contribute most in favour of lineage II (positive peaks). Thus, the observed segment-wide mass difference is already partially explained by a small set of highly lineage-informative positions.

Finally, to determine which amino acids are responsible overall, we summed the mass-weighted matrix across all positions to obtain a total contribution for each residue (Fig. 3E, right). Negative values indicate residues that make lineage III heavier (more frequent and/or used in heavier sequence contexts in lineage III), whereas positive values indicate residues that make lineage II heavier. This decomposition shows that the higher average molecular mass of lineage III is driven predominantly by increased usage of arginine (R) and glutamine (Q), which have the largest negative contributions to (II − III), followed by threonine (T), isoleucine (I), glutamate (E), and phenylalanine (F). Conversely, enrichment of valine (V) and alanine (A), and to a lesser extent aspartate (D), histidine (H), proline (P), lysine (K), and leucine (L), in lineage II shifts mass in the opposite direction and partially offsets this effect. Grouped by chemistry, lineage III preferentially uses heavier polar and some charged residues (R, Q, T, E), as well as some bulky hydrophobic residues (I, F), whereas lineage II is relatively enriched in relatively lighter aliphatic residues (V, A, L, P) together with selected charged residues (D, K, H). The overall sum of these contributions is approximately −179.4 (II−III). In other words, the mean lineage III GPC is ≈180 Da heavier than the mean lineage II GPC, primarily because lineage III has shifted towards heavier amino acids at a subset of positions. Thus, the mass difference between these lineages is not caused by a single dominant substitution, but by a lineage-specific shift in amino-acid usage concentrated at a limited set of positions.

### Population-Scale Structural Modelling of 613 LASV GPC sequences using AlphaFold2 identifies structural positioning of the indel

To assess the structural consequences of the N-terminal indel that underlies the 490/491-aa length polymorphism in GPC, we modelled LASV GPC in its prefusion conformation using AlphaFold2 Multimer v2.3 (ColabFold implementation)^42^. Structurally, the indel position can be assigned to the GP1 domain. During the fusion process, the GP1 domains dissociate from the GPC, which makes the structure modelling of the postfusion conformation obsolete. In doing so, we generated a comprehensive catalogue of LASV glycoprotein structures across lineages. Together, these models capture both intra- and inter-lineage diversity and provide a resource for comparative analyses of structural variation, stability, and potential immunogenic differences among LASV lineages.

We generated structural models for all viable LASV GPC sequences included in this study. The dataset covers five major LASV lineages, comprising 378 models from lineage II, 157 from lineage IV, 53 from lineage III, 13 from lineage VII, and 8 from lineage V. In addition, we modelled four sequences representing less well-defined or apparently extinct lineages: KM822128 (lineage I, Pinneo strain), KT992425 (lineage VI, Kako strain), MG812675 (lineage I), and MK107927 (lineage VI or unclassified). The generation of a comprehensive structural dataset has several benefits. First, the structural effect of point mutations can be assessed on a population scale. Second, it accounts for the massive intralineage diversity within LASV^11^.

Initial predictions generated without template guidance yielded low-confidence models, so we incorporated structural templates to improve accuracy. We tested two template strategies. First, we used LASV GPC as a template, taking advantage of experimentally determined lineage IV structures (PDB: 7PUY^8^). Second, we used the Lujo virus (LUJV) GPC in the prefusion conformation (PDB: 8P4T^43^) as a more generic arenavirus reference that is more distant from Old World clades. We reasoned that these template choices may guide AlphaFold2 towards more reliable models while minimizing bias toward any one LASV lineage or arenavirus subgroup.

Using the LASV-based template, AlphaFold2 model 2 slightly outperformed the other four models in terms of pLDDT, pTM, and ipTM scores (see Methods), with mean values of approximately 85, 0.84, and 0.83, respectively. All models were predicted with the stable signal peptide (SSP) present; however, the pLDDT of this domain was consistently low. Analysis of local pLDDT values within the N-terminal region of GP1 showed reduced AlphaFold2 confidence for residue 59 when the insertion (i.e., the 491-aa form, with residues present at both positions 60 and 61) was present (Fig. 5A). This local dip in confidence is consistent around the SSP–GP1 junction in sequences carrying the indel.

**Fig. 4:**
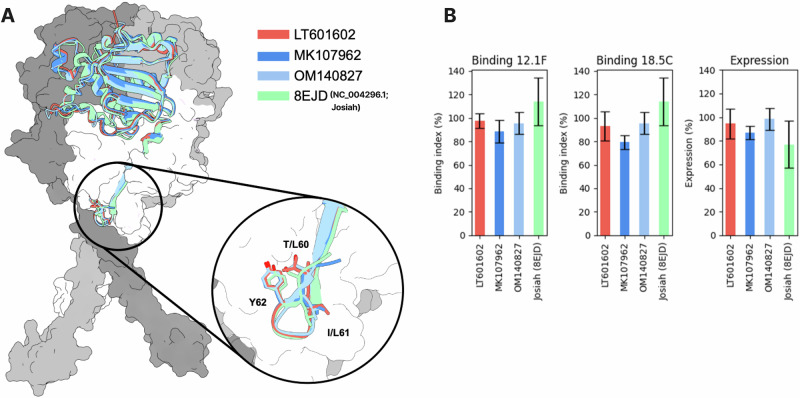
Population-scale structural assessment of LASV GPC across lineages and evaluation of the N-terminal indel. **A** Representative prefusion GPC model with and without the N-terminal “insertion” in GP1. Four structures are shown: two models containing the “insertion” (GenBank ID: MK107962; PDB: 8EJD) and two models lacking the insertion (GenBank IDs: LT601602 and OM140827). Four representative GP1 domains are depicted in cartoon style, and the rest are represented as a surface. **B** Expression and antibody binding of selected GPC variants. Shown are GPC expression levels and binding indices for dimer-binding (18.5 C) and monomer-binding (12.1 F) LASV-specific antibodies, as measured by flow cytometry.

In contrast, using the LUJV template resulted in overall lower prediction confidence, with mean pLDDT values of ~70 (pTM = 0.75, ipTM = 0.74). One possible explanation is the different performance trend across recycle iterations: with the LASV template, prediction accuracy improved markedly during the first recycles, whereas with the LUJV template, more recycles were required to achieve comparable quality metrics. Similar to the LASV-based predictions, AlphaFold2 displayed reduced confidence around residue 59, and the per-residue pLDDT profiles were broadly comparable between the LASV- and LUJV-based models (Fig. 4A).

**Fig. 5:**
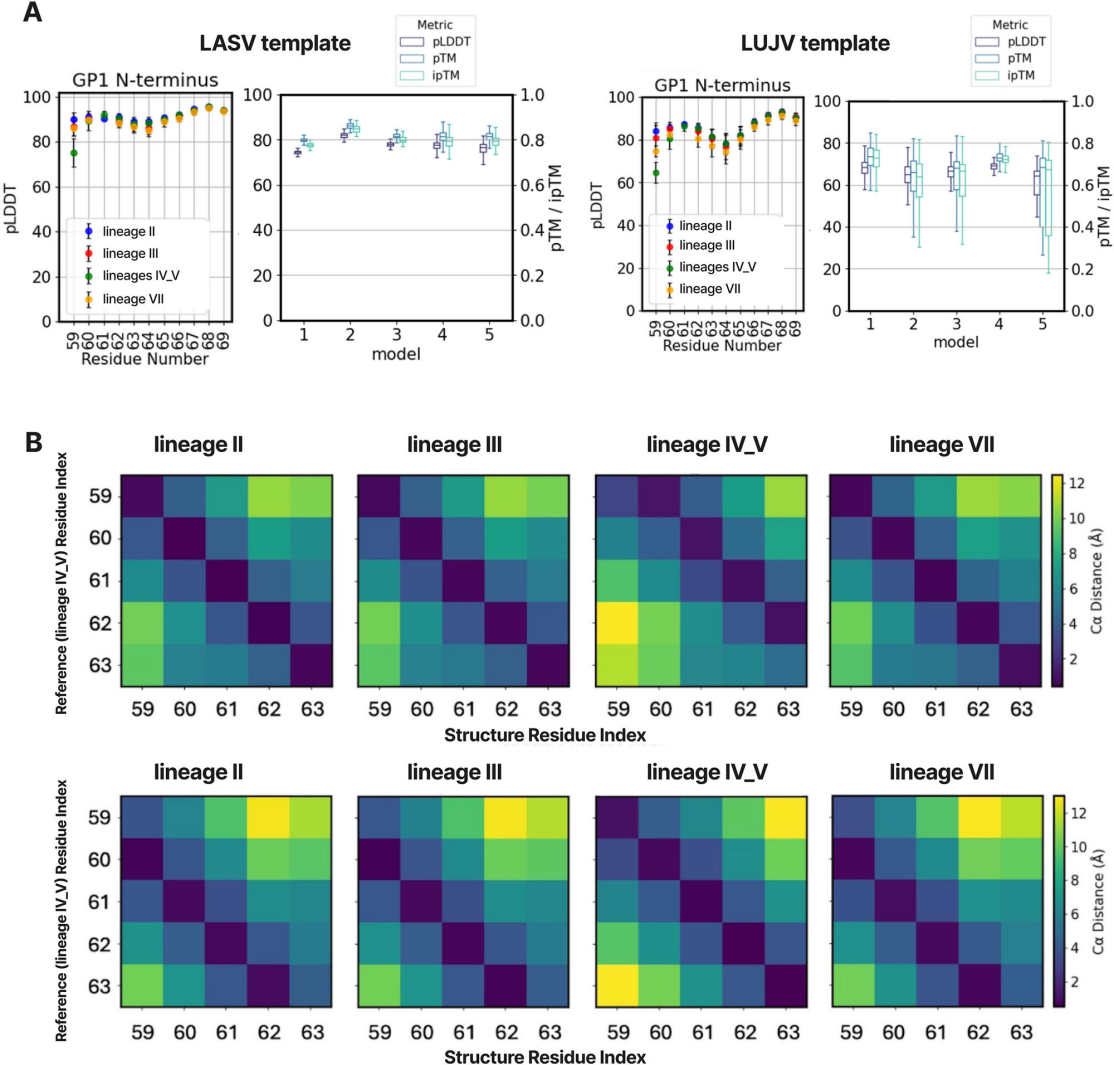
Population-scale structural assessment of LASV GPC across lineages and evaluation of the N-terminal indel. **A** Performance of AlphaFold2 Multimer (AF2) structure predictions for LASV GPC using either LASV GPC (PDB: 7PUY) or LUJV GPC (PDB: 8P4T) as a template. For each template, the left panels show average per-residue pLDDT values for the N-terminal region of the GP1 domain, and the right panels summarise global confidence metrics (pLDDT, pTM, and ipTM) across all models. **B** Structural location and impact of the insertion/deletion in GP1. Heatmaps show average Cα–Cα distances between homologous residues in GP1 domains from different lineages. Each panel compares all models of a given lineage (columns) to a reference lineage (top row: lineage II; bottom row: lineage IV/V). Distances are averaged across all predicted structures for each lineage.

For all structural models that did not include the indel, we introduced a gap at position 61 in the pLDDT plots, reflecting the presumed site of the indel. However, initial analyses indicated that AlphaFold2 is not sensitive to this adjustment at the sequence-annotation level; instead, the algorithm effectively extends the longer chains from the N-terminus (Fig. 5B). We validated this behaviour by analysing Cα–Cα distances across residues in the region for all models, confirming that the effect was consistent across the dataset.

Comparative analysis of representative structural models with (GenBank ID: MK107962, PDB accession: 8EJD) and without (GenBank IDs: LT601602, OM140827) the “insertion” suggests that this indel does not substantially alter potential interactions within the GP1 domain beyond residue 62 (Fig. 4A). In all cases, the conserved tyrosine at position 62 remains oriented toward the GP2 domain. Instead, the inserted residue projects towards the solvent or membrane, indicating a limited impact on overall GPC prefusion stability.

### Experimental testing to GPC length polymorphism tolerance

To test whether the N-terminal length polymorphism and associated sequence differences affect GPC expression or antibody recognition, we selected representative viral GPCs carrying either the 490- or 491-amino-acid form and expressed them in Expi293 cells. The following GenBank constructs were used: LT601602, MK107962 (lineage II, without indel), OM140827 (lineage II, with indel), and Josiah (lineage IV, reference strain; PDB ID: 8EJD). All constructs contained an intracellular C-terminal Twin-Strep tag, allowing us to quantify surface expression and antibody binding by flow cytometry. GPC expression was measured by staining the intracellular tag with PE-labelled Strep-Tactin®XT, and antibody binding was assessed using a dimer-binding antibody (18.5 C) and a monomer-binding antibody (12.1 F), followed by a secondary antibody directed against the human Fc (fragment crystallizable) region. Antibodies 12.1 F and 18.5 C were chosen because they are conformation-sensitive and only bind when the GPC is expressed in its prefusion conformation. Specifically, 18.5 C binds to residues that correspond to the GP1 N terminus and the T-loop, and 12.1 F binds at the apex of the GP1 domain^44^.

Across the tested constructs, we observed only modest quantitative differences in both expression and binding index, with no clear evidence for a consistent, large effect of the indel on these readouts (Fig. 4B). Accordingly, the expression levels are similar to each other with 94.4, 86.8, 98.3, and 77 for LT601602, MK107962, OM140827, and Josiah, respectively. This also translates to the antibody binding indices. Here, a binding index ranging from 88.4 to 113% could be detected for the different constructs, a trend that could also be measured for 18.5 °C, where the values ranged from 79 to 114.3%. In other words, GPC variants with and without the indel behaved similarly in this cellular assay. These data suggest that the N-terminal indel and the additional sequence differences between the selected reference GPCs are tolerated to a reasonable extent and do not grossly perturb GPC expression or recognition by these antibodies.

## Discussion

More than fifty years after its discovery, Lassa fever continues to cause recurrent epidemics across West Africa and sporadic exported cases, yet no licensed vaccine or LASV-specific antiviral is currently available^1–4,6,7^. Despite extensive virological and genomic work, the biological factors that contribute to differences in virulence, transmissibility, and immune recognition among LASV lineages remain poorly understood. LASV lineages differ in clinical outcomes and immunogenicity—lineage VII strains can be more pathogenic than lineage II in non-human primates^17^, lineage I is unusually resistant to many neutralizing antibodies^19^, and survivor plasma shows lineage-biased neutralization^20,21^. Recent reverse-genetics work has further implicated the L protein as a key viral determinant of pathogenic differences between closely related LASV strains^36^. However, the fundamental characteristics of LASV that may explain these phenotypic differences, particularly at the level of basic protein properties, have remained largely undefined. Put simply, whether LASV lineages are equivalent or different in their physical properties, especially at the population scale, remains an open question with implications for hypothesis generation and future comparative analyses.

Here, we show that LASV lineages differ measurably in the physical properties of their proteins at the population scale. By analysing all four LASV proteins across lineages, we identify systematic differences in sequence length, theoretical molecular mass, and amino-acid composition. An especially notable finding concerns the S segment, where lineage III consistently encodes heavier GPC and NP than lineage II despite identical or nearly identical protein lengths, whereas the L-segment proteins (RNAPOL and Z) display a more heterogeneous pattern: RNAPOL shows tightly clustered but lineage-structured length distributions (with shorter sequences predominant in lineage III and lineage VII encoding the longest polymerase), and Z is almost invariant in length but exhibits lineage-specific differences in molecular mass and pronounced proline enrichment, particularly in lineages IV, V, and VII. One clear result from our analysis is that the LASV nucleoprotein is 569 amino acids in length across all lineages, establishing a fundamental reference point against which future deviations may be readily identified. Length differences observed here are caused by in-frame codon indels. Frameshift indels likely also occur but would be expected to generate non-viable viruses; hence, only a subset of indel events are compatible with long-term persistence and spread and thus become visible at the population level.

In this way, we extend prior work on codon-usage bias^12,27^ by showing that codon-usage differences are accompanied by subtle but quantifiable changes in amino-acid usage. Given that the L protein has been implicated as a key viral determinant of pathogenic variability^36^ and that proline-rich late-domain motifs in Z are critical for virus-like particle budding and efficient particle release^37–40^, the segment- and lineage-specific mass and composition patterns we observe may reflect fine-tuned adaptations in replication, assembly, or budding. Notably, the increased proline usage observed in lineage VII (see results) raises the possibility that such compositional differences may contribute to lineage-specific biological properties, including the increased virulence reported for this lineage^18^. Whether the measurable physical differences we describe help explain lineage-specific biological behaviours—for example, differences in tissue tropism, replication kinetics, or immune recognition—will require targeted functional experiments, such as swapping L or Z proteins between lineages, editing specific late-domain residues, or deploying reverse-genetics approaches^36^. Meanwhile, our observations on protein length and theoretical molecular mass may directly benefit sequencing, protein-focused studies, and public health by providing expected lineage-specific protein features and facilitating the detection of unexpected results.

A central finding is that the mass difference between lineage II and lineage III GPC is not driven by a single “special” mutation, but by a distributed, lineage-specific shift in amino-acid usage. Using three complementary metrics—random forest feature importance, Manhattan distance, and Pearson correlation—we show that 177 of 491 GPC alignment positions contribute measurably to this mass difference (Fig. 3E), with the remaining sites being effectively indistinguishable in their amino-acid distributions. Within this subset, a small number of positions contribute disproportionately to the mass difference, with position 76 emerging as the largest single contributor in favour of lineage III and position 44 in favour of lineage II. Notably, position 76 has previously been highlighted as an immunologically important site in LASV GPC, affecting antibody 21.10c and featuring in epitope mapping and deep mutational scanning analyses^31,32^. Decomposition of the mass difference by residue further reveals that lineage III is enriched for heavier polar and some charged residues (R, Q, T, E) and some bulky hydrophobic residues (I, F), whereas lineage II preferentially uses lighter aliphatic residues (V, A, L, P) and selected charged residues (D, K, H). The net effect is that the mean lineage III GPC monomer is approximately 180 Da heavier than the mean lineage II GPC, largely because lineage III has shifted toward heavier amino acids at a limited set of positions. This quantitative, mass-weighted compositional analysis provides a mechanistic explanation for the segment-wide mass differences observed at the protein level.

Lineages II and III both circulate in Nigeria, but in largely non-overlapping regions: lineage II predominates in the south-central and southwestern states, whereas lineage III is found mainly in the north-central belt^11^. These regions differ in human population structure and ancestry, with Hausa/Fulani populations more common in the north and Yoruba/Igbo and related groups more common in the south. It is therefore plausible that GPC—the viral protein that engages α-dystroglycan and LAMP1 and thus sits at the interface of virus and host^6,8^—may be undergoing fine-tuned adaptation to host backgrounds that differ in glycosylation patterns or other immunogenetic factors. This would be consistent with evidence that host genetic variation, including variants at the *LARGE1* locus modulates susceptibility and outcome of Lassa fever in Nigeria^45^. However, this remains a hypothesis: while our data demonstrate robust lineage-specific shifts in amino-acid usage, causal links between these shifts, specific host genetic backgrounds, and clinical phenotypes will require integrated viral–host association studies and experimental validation.

We also asked whether the N-terminal GPC length variation, arising from an indel around residues 60–61, results in functionally relevant divergence between lineages. Earlier studies noted lineage-specific gaps or codon differences near this region in a small number of sequences or constructs^19,22,23,30,31^, but could not assess their prevalence or structural impact at the population scale. Our alignment- and phylogeny-based analyses show that this short indel explains the 490/491-aa length polymorphism and is strongly enriched in lineage II, with rare “long” variants and outliers in specific subclades (Fig. 2). In our alignment framework, the indel in lineage II maps most consistently to position 61, whereas several previous studies described a gap at position 60; this discrepancy likely reflects differences in alignment strategy and register rather than fundamentally different biology, and highlights the value of population-scale, phylogeny-aware alignments for precise positional mapping. Precise positional mapping is essential to ensure that subsequent laboratory analyses target the intended sites and to avoid the unintentional construction of LASV variants that do not occur in nature.

AlphaFold2 Multimer modelling, using both LASV and LUJV GPC templates^8,42,43,46^, indicates that the overall prefusion architecture of GPC is highly conserved across lineages and length variants. The conserved tyrosine at position 62 remains oriented toward GP2 in all models, and position 61—the indel site—tends to project toward the solvent or membrane rather than interfering with the GP1–GP2 interface. Cα–Cα distance analyses further indicate that the indel does not globally remodel GP1 structure and is positioned at the extreme N terminus (around residue 59) rather than producing a structural perturbation centred at position 61. Taken together, these observations suggest that the indel represents a tolerated local variation at the individual level rather than a major functionally relevant modification. Nevertheless, this example emphasizes the importance of investigating proteins not only at the sequence level but also in structural context. Meanwhile, It should be noted that some bias may arise from the choice of templates, which may influence the interpretation of insertion/deletion or mutation effects.

Consistent with this structural tolerance, our functional experiments show no large differences in expression or antibody binding between representative GPCs with and without the indel. In a flow cytometry assay, all variants tested exhibited similar expression levels and binding indices for a dimer-preferring GPC-B antibody (18.5 C) and a GP1-A monomer-binding antibody (12.1F), both of which have been structurally and functionally characterized in previous work^9,31,47,48^. Although modest quantitative differences were observed, there was no clear pattern indicating that the 490- versus 491-amino-acid forms differ substantially in their ability to be expressed or recognised by these antibodies. Together with the structural modelling, these data suggest that both the N-terminal indel and the broader pattern of compositional shifts are compatible with a stable GPC fold and do not grossly perturb the epitopes recognised by the tested monoclonal antibodies. This does not exclude more subtle functional or immune effects—particularly in the context of polyclonal responses or in vivo infection—but it argues against a simple binary model in which one length class is dramatically more or less “fit” than the other.

Methodologically, our study provides a general framework for dissecting lineage-specific molecular diversity. By combining population-level amino-acid composition analysis, mass-weighted difference profiling, machine-learning–based feature importance, and population-scale structure prediction, we move from descriptive statements about “lineage diversity” to quantitative decompositions that identify which properties, residues, and positions contribute most to lineage separation. This approach is not limited to LASV and could be applied broadly to population-scale genomics whenever multiple subgroups or lineages are compared within a shared alignment—for example, drug-resistant versus drug-sensitive strains, different host species or reservoir populations, or viral variants associated with distinct clinical outcomes.

Finally, in the process of answering our questions for this study, we provide a comprehensive, lineage-resolved catalogue of LASV GPC structures predicted at the population scale. These models cover all major lineages and capture both intra- and inter-lineage diversity in a format that is readily amenable to comparative structural analysis, epitope mapping, and in silico vaccine design^10,31^. Experimental generation of LASV GPC structures has been reported to be challenging^48^. We therefore anticipate that our predicted structural catalogue will help address this gap by providing broad and unprecedented access to LASV GPC structural diversity at scale, complementing existing experimentally determined structures^8,31^ and the limited number of previously reported predicted LASV structures^49^. We envisage that such a resource will be valuable for designing broadly neutralising antibody cocktails, identifying lineage-specific vulnerabilities, and rationally selecting GPC variants as candidate immunogens. Our results further support the idea that future vaccine strategies may need to account for lineage-specific molecular features—either by explicitly including multiple lineage representatives in multivalent formulations or by designing immunogens that focus immune responses on conserved, structurally constrained regions of GPC.

This work has several limitations. First, it is constrained by the available sequence data, which remain sparse and geographically biased relative to the true burden and diversity of LASV^11,12,33^. Second, our structural conclusions rest primarily on predicted models rather than experimentally determined structures for all lineages and variants, and it has been shown that AlphaFold2 does not fully capture dynamic or glycan-mediated effects^8,31,46,50^. Relatedly, our mass analyses do not account for post-translational modifications, including glycosylation; experimentally derived molecular masses for the glycosylated GPC monomer have been reported to be around 82 kDa^51^. Third, our functional characterisation focuses on a limited set of monoclonal antibodies and a single cell system, and therefore cannot exclude more subtle or context-dependent functional differences, particularly in polyclonal immune responses or in vivo. Nevertheless, by quantitatively linking population-scale sequence variation, amino-acid usage, molecular mass, structure, and experimental phenotypes, our study provides new insight into how LASV lineages differ at the molecular level and offers a foundation for interpreting LASV behaviour, benchmarking protein properties, and guiding future mechanistic studies and rational vaccine and therapeutic design.

## Methods

### The GPC data

From our previous work, CLASV^52^, we obtained a curated and aligned dataset of Lassa virus (LASV) glycoprotein (GPC) amino acid sequences (*n* = 753). Sequences with more than one missing amino acid were excluded, leaving 674 sequences with ≤1 missing residue, of which 44 still contained one missing amino acid. Incomplete sequences with alignment gaps at either the 5′ or 3′ termini were also filtered out, resulting in 645 complete sequences.

Lineage assignments followed the CLASV training annotations^52^, consistent with the lineage classification scheme used in our Nextstrain Lassa dataset (https://nextstrain.org/lassa/gpc?c=gpc_lineage)^53^. Within the IV/V grouping, lineage V was separated from lineage IV by applying a branch-based split consistent with Manning et al.^13^.

Due to limited sequence representation, lineages I and VI were excluded from downstream analyses. The final dataset comprised 641 GPC sequences, distributed as follows: lineage II (*n* = 396), lineage IV (*n* = 165), lineage III (*n* = 56), lineage VII (*n* = 13), and lineage V (*n* = 11).

### The Nucleoprotein data

Nucleoprotein (NP) sequences corresponding to the CLASV GPC dataset (*n* = 753) were retrieved from the LASV coding region dataset downloaded from NCBI on 24 September 2025. Sequences annotated as “nucleoprotein” in their description fields and sharing GenBank accessions with the GPC dataset were retained, yielding 716 sequences.

Sequences were aligned using the MAFFT online server^54^ with default parameters (https://mafft.cbrc.jp/alignment/server/). Incomplete sequences with alignment gaps at either terminus were removed, leaving 679 complete sequences. Sequence handling, visualisation, and nucleotide-to-amino-acid conversion for NP and the other protein-coding genes were performed using AliView^55^ and Biopython^56^. Sequences with more than one missing amino acid were excluded, resulting in 638 sequences with ≤1 missing residue, 13 of which still contained one unknown amino acid. The final filtered dataset contained 634 NP sequences (after excluding lineages VI and I), distributed as follows: lineage II (*n* = 410), lineage IV (*n* = 155), lineage III (*n* = 55), lineage VII (*n* = 13), and lineage V (*n* = 1). Lineage V was retained despite limited representation to maintain lineage consistency across analyses.

### The RNA Polymerase data

For the RNA-dependent RNA polymerase (L segment), 614 samples were identified from the CLASV dataset^52^ as GPC sequences that had corresponding L segment data based on our live Nextstrain trees (https://nextstrain.org/lassa). A total of 574 sequences annotated as “polymerase” were retrieved from the LASV coding region dataset and aligned using MAFFT^54^ with default settings.

Upon inspection of the alignment in Aliview, six sequences (OM791227, MK117845, MK107886, MK107845, KF478764, and KF478761) were removed due to poor alignment quality, leaving 568 sequences. Sequences with incomplete lengths or flanking gaps were removed, leaving 499 sequences. After excluding those with more than one missing amino acid, 418 sequences remained, of which five still contained one ambiguous residue (X). To reduce noise, sequences with unique lengths were excluded, resulting in 412 final (excluding LVI and LI) RNAPOL sequences distributed as: lineage II (*n* = 291), lineage IV (*n* = 84), lineage III (*n* = 32), lineage VII (*n* = 3), and lineage V (*n* = 2).

Lineage annotation was performed using our Nextstrain LASV L-segment dataset (https://nextstrain.org/lassa/l?c=l_lineage&tl=genbank_accession)^53^.

### The Z protein data

For the Z matrix protein, 562 sequences were retrieved from the same LASV coding region dataset that also had representatives in the CLASV GPC set^52^. After alignment with MAFFT^54^ and removal of incomplete sequences, 509 remained. Following exclusion of sequences with more than one ambiguous amino acid, 495 sequences were retained, 21 of which still contained one missing amino acid.

After excluding sequences belonging to lineages I and VI, a final total of 492 Z protein sequences were included in the analysis.

### Calculation of protein sequence properties

Protein biophysical properties were computed with Biopython (Bio.SeqUtils.ProtParam.ProteinAnalysis)^56^.

Sequence lengths and measured properties (e.g., molecular mass, amino-acid counts) were assembled into a dataframe with lineage annotations and sorted by lineage for visualization. Amino-acid composition per sequence was computed as the proportion of each residue (count of AA/total residues). To assess whether composition and biophysical properties differ by lineage, we summarized per-lineage means (±SD), visualized heatmaps, and radar plots of mean compositions.

### Identifying lineage dependent amino acid differences

While Shannon entropy^57^ is traditionally used to detect variable regions in multiple sequence alignments, it does not distinguish which specific alignment positions differ between two defined groups of sequences. In this study, our goal was not simply to locate highly variable regions but to identify positions that consistently differ between two groups of a single alignment on a population scale.

To achieve this, we applied three complementary analytical approaches in a novel way: Manhattan distance, Random Forest (RF), and Pearson correlation analysis to identify positions that differ between two lineages of the LASV GPC: lineage II (*n* = 396) and lineage III (n = 56). These lineages were of particular interest because, although both are major Nigerian lineages, they consistently rank lowest and highest in molecular mass, respectively, across proteins encoded on the S segment (see Results).

### Random Forest analysis for position discovery

To identify specific alignment positions that best distinguish LASV lineages, we analysed the GPC multiple sequence alignment at the residue level. The GPC amino-acid alignment, consisting of *n* sequences and 491 positions, was processed using Biopython’s alignment module. Gaps preceding or following real amino acids were reclassified as “Unknowns” to minimize potential bias from incomplete sequences.

Each amino-acid residue was one-hot encoded into a 21-dimensional vector (representing the 20 canonical amino acids plus a gap). Unknown residues were encoded as zero vectors. Flattening these encodings across the entire alignment produced a feature matrix of size *n* × 10,311 (491 × 21), enabling position-resolved modelling and downstream distance- and correlation-based profiling. Random Forest feature importance was then used to rank informative positions, and these results were complemented with Manhattan-distance profiling and Pearson-correlation analyses to highlight residues showing consistent lineage-associated divergence.

### Feature importance and motif selection

During encoding, each position in the alignment was represented by a flattened 21-element vector, producing 491 × 21 = 10,311 total columns. The Random Forest algorithm generated a feature-importance table containing the column indices and their associated importance scores. To determine which amino-acid position each feature corresponded to, the column index was divided by 21, and the integer part of the quotient was incremented by one (upgrade from zero index).

Feature weights belonging to the same alignment position were summed to yield an aggregated feature-importance score per position. These scores were then ranked from highest to lowest, identifying residues that contributed most strongly to lineage discrimination. Each high-ranking position corresponds to a specific site in the GPC alignment that may encode functionally relevant or lineage-specific motifs.

#### Manhattan distance and Pearson correlation analyses

To further quantify position-specific compositional differences between groups, we computed both Manhattan distance and Pearson correlation coefficients on normalized amino acid count matrices.

The GPC alignment (n × 491) was divided into two groups based on lineage—lineage II and lineage III. For each group, amino-acid counts were computed per alignment position, yielding two 21 × 491 matrices (20 amino acids plus gap). Gaps occurring before or after real amino acids were converted to “Unknowns,” and counts of “Unknowns” were added to the most frequent variant at each position to account for missing data.

Each matrix was normalized by dividing amino-acid counts at every position by the total number of sequences in that group, resulting in two relative-frequency matrices (A and B). The absolute compositional divergence between two matrix columns A_j_ and B_j_ was then computed using the normalized Manhattan distance:

$$d(j)=\frac{1}{2}L1(j)=\frac{\sum {|A}(i,j)-B(i,j)|}{2}$$

where *i* ranges from 1 to 21 (the amino acids plus gap), and *j* represents the position in the alignment. This metric was used to rank positions by the magnitude of compositional difference between the two lineages, with the highest distances corresponding to the most distinct sites.

Next, Pearson correlation coefficients were computed using the same normalized matrices. For each alignment position *j*, we calculated the correlation between amino-acid frequencies across the two lineages:

$$r(j)={corr}({A}_{j},{B}_{j})$$

This produced a vector of correlation values representing the degree of compositional similarity between groups at each site. Positions with low correlation values indicate divergent amino-acid variability between the two lineages. The resulting vector was ranked by absolute correlation values, with the lowest correlations marking positions of greatest divergence.

### Investigating molecular mass differences across lineages

To investigate which positions and residues drive the molecular mass difference between lineage II and lineage III, we first computed, for each alignment position, the difference in normalised amino acid frequencies between the two groups (lineage II − lineage III), using the same normalised matrices described above. We then removed all alignment columns in which the two lineages had identical amino acid usage, yielding a reduced 21 × 177 matrix (21 amino acid/gap states by 177 informative positions).

To identify which positions contribute most strongly to the mass difference, we multiplied each amino acid row by its molecular mass, so that each cell represents the mass-weighted frequency difference for that residue at that position. Summing these values across rows for each column provided a per-position mass difference profile, from which we ranked positions by their absolute contribution to the overall mass shift between lineages.

Conversely, to quantify the total contribution of each amino acid, we summed the mass-weighted matrix across columns for each row. This yielded a per–amino acid contribution score, where positive values indicate residues that make lineage II heavier and negative values indicate residues that make lineage III heavier. Together, these analyses allowed us to decompose the global mass difference into contributions from specific positions and amino acids.

### Random forest analysis for AAC difference test

Given population-scale measurements of amino acid composition across Lassa virus lineages, an important question is whether these compositional profiles differ significantly among lineages. A straightforward way to assess this is to determine whether a machine-learning classifier can distinguish the sequences based solely on their amino-acid composition. If the classifier performs well, this suggests that compositional patterns are lineage-specific and biologically meaningful.

Target variables were one-hot encoded using the pandas.get_dummies() method, with “lineage” serving as the classification target. The dataset was then divided into training (80%) and testing (20%) subsets using scikit-learn’s train_test_split function, applying a random state of 42 and stratifying by lineage.

Random Forest (RF) classifiers were implemented with scikit-learn’s RandomForestClassifier, following the framework described in Daodu et al.^52^. The random state for the RF model was set to 80, and the seed state to 42 to ensure reproducibility. Model performance was evaluated using the classification_report() function, which computes precision, recall, and F-score on the test set. The model’s ability to classify sequences by lineage based only on their compositional features served as an indicator of whether statistically separable amino-acid usage patterns exist across Lassa virus lineages.

### Phylogenetic analysis of GPC

From our previous work on CLASV^52^, we obtained a curated dataset of GPC amino acid sequences (*n* = 753). Sequences containing any missing amino acid residues were removed, leaving 613 complete sequences. These were aligned at the amino acid level using the MAFFT online tool (https://mafft.cbrc.jp/alignment/server/index.html) and then reverse-translated to nucleotide sequences using custom scripts. For each sequence, codons were assigned according to the corresponding amino acids in the original nucleotide sequence of that sample, and amino acid gaps in the alignment were represented as codon gaps in the nucleotide sequences.

Using a pipeline based on Snakemake^58^, Nextstrain^59^, and Augur^60^, we reconstructed both a Maximum Likelihood tree and a time tree. The alignment and metadata, which include sampling dates, country, and host information, are the inputs to the pipeline. A Maximum Likelihood tree was built using IQ-TREE^61^ with the application programming interface (API) provided by Augur. Similarly, using the Augur API, the tree was processed by TreeTime^62^, along with the metadata, to generate a time-calibrated tree.

The trees and accompanying metadata were parsed using the Augur export command into a JSON file, which was subsequently visualized using Auspice (available at https://auspice.us/), a part of the Nextstrain^59^ toolkit. All phylogenetic images were generated using Auspice—which has been edited for clarity in Figma (available at https://www.figma.com/).

### LASV GPC structure prediction

The same 613 gap-free complete sequences as above were used for structural modelling. Because the LASV GPC forms a trimeric assembly, each sequence was prepared by concatenating three monomeric subunits according to the known domain architecture of the glycoprotein. The subunits were defined as follows: the stable signal peptide (SSP; residues 1–58), glycoprotein subunit 1 (GP1; residues 59–258), and glycoprotein subunit 2 (GP2; residues 259–491 - the end). For each sample, the concatenated construct used for modelling followed the pattern:

*final_seq = f*″*{ssp}:{gp1}:{gp2}:{ssp}:{gp1}:{gp2}:{ssp}:{gp1}:{gp2}*″*.replace(*′*-*′, ″*)*.

This configuration reflects the biological trimeric state of GPC while removing alignment gaps introduced during multiple sequence alignment. Each sequence was then saved individually in FASTA format, using its sample ID as the filename. The full processing and modelling pipeline, including sequence preparation and parameter configuration, is available in the GitHub repository (see data availability).

We performed structural predictions using the ColabFold implementation of AlphaFold2-Multimer v2.3^42,46^. Because de novo prediction of viral glycoproteins without templates can be challenging, template structures were systematically included (PDB: 7PUY^8^ and PDB: 8P4T^43^). We used AlphaFold2 because custom template handling is easily adaptable, allowing us to explore the impact of template selection in this study. Two complementary prediction procedures were employed to enhance model confidence. One prediction run facilitated LASV GPC as a template, taking advantage of experimentally determined lineage IV structures (PDB: 7PUY^8^). A second prediction run used the Lujo virus (LUJV) GPC in the prefusion conformation (PDB: 8P4T^43^). Structure refinement was performed using AMBER relaxation^63^, and the number of recycles was increased to 20 to improve structural convergence. All other parameters were maintained at their recommended default values^46^.

The quality of the predicted models was evaluated using standard AlphaFold2 metrics, including the predicted Local Distance Difference Test (pLDDT) score, predicted Template Modelling (pTM) score, and interface predicted Template Modelling (ipTM) score. These metrics provided internal benchmarks for assessing overall model accuracy, inter-subunit interface quality, and confidence in local structural regions.

Structural models were visualized and analyzed in UCSF ChimeraX^64^, which allows high-resolution rendering and detailed examination of protein structures.

### GPC expression and flow cytometry analysis

We selected four LASV strains to investigate indel effects: LT601602 (lineage VII, derived from secondary transmission in Germany^3^, MK107962 (lineage II, without indel), OM140827 (lineage II, with indel), and Josiah (lineage IV, reference strain; PDB ID: 8EJD). Expression levels of the different LASV GPC variants were assessed by a flow cytometry assay. To this end, LT601602, MK107962, OM140827, and Josiah (8EJD) constructs were designed with a furin cleavage site, instead of S1P, and a C-terminal intracellular His and TwinStrep tag and ordered from Integrated DNA Technologies (Newark, New Jersey, USA). The constructs were expressed transiently in Expi293 cells (ThermoFisher, Waltham, Massachusetts, USA) by transfection with PEI. Per 3 million cells, 1ug DNA was mixed with 3ul of PEI in 70ul OptiMem (ThermoFisher, Waltham, Massachusetts, USA) and incubated for 15 minutes at room temperature. Following the DNA-PEI-medium mix was added drop-wise onto the cells. Cells were harvested after an incubation period of 48 h using centrifugation at 300xg and stained as described previously following the protocol in Peter et al.^65^; Irrgang et al.^66^. All washing steps and dilutions were carried out in FACS buffer (PBS with 0.51% BSA). After each staining step, cells were washed by adding 200 µL of FACS buffer followed by centrifugation at 300 × *g* for three cycles. Unless otherwise specified, all incubations were performed for 30 minutes at 4°C in the dark. The cells were stained extracellularly with 10ug/ml of 12.1 F or 18.5 C, both LASV-specific antibodies^6^. These were produced and purified as described previously in Barden et al. (2024) by transient transfection of Expi293 cells (ThermoFisher, Waltham, Massachusetts, USA). Following, the cells were incubated with a secondary Alexa Fluor 647 conjugated goat anti-human IgG antibody (#AB_2337880, Jackson ImmunoResearch, West Grove, Pennsylvania, USA) diluted 1:400. Then the cells were fixed with 2% PFA in PBS and permeabilized with FACS buffer with 0.5% Saponin for 10 minutes. Then, the intracellular Strep-Tactin Tag was detected with PE labelled Strep-Tactin^®^XT (#6-5400-001, iba, Göttingen, Germany) diluted 1:400. After another washing step. The cells were analyzed using a NovoCyte Quanteon (Agilent, Santa Clara, California, USA) and FlowJo (FlowJo LLC, Ashland, Oregon, USA). The binding index was calculated using the following formula:

$${Binding}\,{index}=(( \% {IgG}\,{positive}\,{cells}\times {MFI}\,{of}\,{IgG}\,{positive}\,{cells})/( \% {Strep}-{Tactin}-{positive}\,{cells}\times {MFI}\,{of}\,{Strep}-{Tactin}\,{positive}\,{cells}))\times 100$$

## Data Availability

Structural models generated in this study are available at Zenodo: 10.5281/zenodo.17642142. Raw and intermediate data to reproduce the results are available on GitHub: https://github.com/JoiRichi/lasv_extra_data/.

## Code availability

Scripts and analysis workflows are available on GitHub: https://github.com/JoiRichi/lasv_extra_data/.
